# The Occupational and Environmental Respiratory Exposome as a Potential Modulator of Adaptive Resistance to EGFR and ALK Inhibitors in Non-Small Cell Lung Cancer

**DOI:** 10.3390/cancers18091364

**Published:** 2026-04-24

**Authors:** Irina Luciana Gurzu, Claudia Mariana Handra, Cristina Mandanach, Nina Ionovici, Bogdan Gurzu

**Affiliations:** 1 Department of Preventive Medicine and Interdisciplinarity, Discipline of Occupational Health, Grigore. T. Popa University of Medicine and Pharmacy, 700115 Iasi, Romania; 2 Clinical Department V, Carol Davila University of Medicine and Pharmacy, 050474 Bucharest, Romania; claudia.handra@umfcd.ro (C.M.H.); cristina.paraschiv@drd.umfcd.ro (C.M.); 3 Occupational Medicine Department, University of Medicine and Pharmacy of Craiova, 200349 Craiova, Romania; nina.ionovici@umfcv.ro; 4 Department of Morfofunctional Sciences II, Faculty of Medicine, Grigore T. Popa University of Medicine and Pharmacy, 700115 Iasi, Romania; bogdan.gurzu@umfiasi.ro

**Keywords:** exposome, occupational exposure, EGFR, ALK, non-small cell lung cancer, tyrosine kinase inhibitors, targeted therapy resistance, precision oncology

## Abstract

Background: Targeted therapies directed against oncogenic drivers have substantially improved outcomes for patients with epidermal growth factor receptor (EGFR)-mutant and anaplastic lymphoma kinase (ALK)-rearranged non-small cell lung cancer (NSCLC). Despite high initial response rates, most patients ultimately develop acquired resistance to tyrosine kinase inhibitors (TKIs), reflecting complex biological adaptations under therapeutic pressure. Methods: This narrative review synthesizes experimental, translational, and clinical studies examining how environmental and occupational respiratory exposures may influence resistance mechanisms in EGFR- and ALK-driven NSCLC. The review emphasizes exposure-associated signaling plasticity, inflammatory microenvironmental modulation, metabolic reprogramming, and pharmacokinetic alterations. Results: Recent evidence suggests that respiratory exposures, including cigarette smoke, air pollution, diesel exhaust, and occupational inhalational toxicants, can modulate oncogenic signaling networks relevant to resistance to targeted therapies. These mechanisms include aberrant EGFR activation, bypass signaling through the mesenchymal–epithelial transition receptor (MET) and SRC pathways, epithelial–mesenchymal transition (EMT), adaptive kinome remodeling, and exposure-associated inflammatory signaling, all of which may influence tumor evolution and therapeutic response. Conclusions: This review introduces a novel exposome-driven conceptual framework integrating environmental exposures with signaling plasticity and resistance evolution in oncogene-driven NSCLC. These findings support the concept that the respiratory exposome may represent an underrecognized modifier of targeted therapy response. Incorporating structured exposure assessment into precision oncology approaches may refine risk stratification and inform exposure-aware therapeutic strategies.

## Simple Summary

Targeted therapies against EGFR and ALK have transformed the treatment of non-small cell lung cancer (NSCLC), yet therapeutic responses remain heterogeneous even among patients with similar molecular profiles. While resistance to tyrosine kinase inhibitors has been extensively studied at genomic and signaling levels, the potential influence of environmental and occupational exposures on resistance evolution remains insufficiently explored. This review integrates mechanistic, epidemiologic, and clinical evidence suggesting that chronic respiratory exposures—including cigarette smoke, air pollution, and occupational inhalational toxicants—may modulate tumor signaling networks and therapeutic vulnerability. We propose a conceptual framework in which the respiratory exposome acts as a biological conditioning layer that shapes adaptive resistance to targeted therapies.

## 1. Introduction

### 1.1. Targeted Therapy Revolution in EGFR- and ALK-Driven NSCLC

Targeted therapies directed at oncogenic drivers have fundamentally transformed the management of non-small cell lung cancer (NSCLC). In patients selected based on molecular characteristics, inhibitors targeting epidermal growth factor receptor (EGFR) mutations and anaplastic lymphoma kinase (ALK) rearrangements achieve high response rates and significantly improve survival compared with historical chemotherapy-based treatments [1,2].

EGFR-mutant tumors, most defined by exon 19 deletions and the L858R substitution, demonstrate constitutive receptor activation and pronounced oncogene addiction. This molecular profile confers high sensitivity to EGFR tyrosine kinase inhibitors (TKIs) such as gefitinib, erlotinib, afatinib, and Osimertinib [1,2,3]. These activating mutations are the most frequent actionable alterations in lung adenocarcinoma and provide the molecular foundation for genotype-directed therapeutic strategies in EGFR-driven disease [2,3].

Similarly, ALK-rearranged NSCLC, most often involving EML4–ALK fusion variants, represents a distinct molecular subtype with pronounced sensitivity to ALK inhibitors such as crizotinib, alectinib, brigatinib, and lorlatinib [2,4]. The introduction of successive generations of ALK inhibitors has significantly improved clinical outcomes, particularly by enhancing central nervous system penetration and activity against resistance mutations [4].

Recent genomic analyses have advanced the understanding of lung cancer biology in never-smokers. Large-scale sequencing studies indicate that tumors in never-smokers exhibit distinct mutational processes and environmental signatures compared to smoking-associated cancers, suggesting alternative etiologic pathways related to environmental exposures [5].

Taken together, these advances illustrate the principles of precision oncology, where molecular characterization of tumors increasingly guides therapeutic decision-making in oncogene-driven lung cancer [2].

Despite these therapeutic advances, clinical responses to EGFR and ALK inhibitors remain heterogeneous. This variability suggests that factors beyond tumor genomics contribute to differences in treatment outcomes [2,6].

### 1.2. The Unresolved Problem of Heterogeneous Resistance

Although many patients initially respond to TKIs, most eventually develop acquired resistance typically within 9–18 months of therapy in advanced NSCLC [1,2]. Molecular studies have identified several canonical resistance mechanisms, including secondary EGFR mutations such as T790M or C797S, activation of bypass signaling pathways involving mesenchymal–epithelial transition receptor (MET) or other human epidermal growth factor receptor (HER) family receptors, and histologic transformation to alternative tumor phenotypes [2,7,8].

Resistance to targeted therapies is increasingly understood as an evolutionary process involving dynamic clonal selection rather than a single discrete genetic alteration. Single-cell transcriptomic and genomic analyses have revealed substantial heterogeneity among tumor cell populations exposed to EGFR-TKI therapy, demonstrating that resistant phenotypes can arise through multiple parallel evolutionary trajectories [9]. These studies have also highlighted the importance of drug-tolerant persister cells—rare cellular states that represent approximately 0.1–1% of tumor cell populations and survive initial therapy, subsequently acquiring stable resistance mechanisms [10,11].

Resistance to third-generation inhibitors such as osimertinib is frequently polyclonal. Analyses from the MATCH-R cohort revealed acquisition of additional oncogenic drivers—including rearranged during transfection, ALK, or fibroblast growth factor receptor 3 (FGFR3) fusions as well as KRAS or BRAF mutations—sometimes coexisting within individual resistant tumor cells [12]. Complementary studies using circulating tumor DNA have shown that resistance trajectories may differ across treatment lines and therapeutic contexts, reflecting the dynamic selective pressures imposed by targeted therapies [13].

These findings indicate that therapeutic response reflects interactions between tumor genomic alterations and contextual factors such as microenvironmental signaling and host-related biological influences [10,14].

### 1.3. Beyond Genomics: The Exposome as a Missing Determinant

Beyond tumor-intrinsic genomic alterations, environmental exposures add another layer of biological variability that may influence cancer development and therapeutic response. The exposome, defined as the cumulative measure of environmental exposures throughout the lifespan, provides a framework for integrating non-genetic determinants into models of cancer biology and precision oncology [15,16,17].

In parallel, it is important to recognize that key oncogenic drivers such as EGFR also function as biomarkers beyond NSCLC. Aberrant EGFR expressions and signaling have been implicated in tumor progression and therapeutic response across multiple malignancies, including colorectal and breast cancer. Recent advances in biomaterial-based and biosensing technologies further demonstrate the utility of EGFR as a diagnostic and prognostic biomarker, including electrochemical detection systems and targeted nanocarrier platforms [18,19].

In lung cancer, the respiratory exposome is especially relevant because the pulmonary epithelium is continuously exposed to complex mixtures of inhaled toxicants. Major components include cigarette smoke, environmental tobacco smoke, cooking fumes, ambient air pollution, and occupational inhalational toxicants such as diesel exhaust particles, metals, and particulate matter [20,21,22]. These exposures are well-established contributors to carcinogenesis and may also influence intracellular signaling networks and tumor microenvironmental states that affect therapeutic vulnerability [16,23].

Experimental studies show that environmental pollutants can modulate oncogenic signaling pathways relevant to lung cancer biology. For example, particulate matter and cigarette smoke induce oxidative stress-mediated activation of EGFR signaling, promoting inflammatory mediator release and proliferative signaling cascades [24,25]. Other mechanisms include pollutant-induced EGFR phosphorylation, activation of downstream ERK and STAT pathways, and epithelial–mesenchymal transition (EMT) triggered by toxic metals such as cadmium [26,27].

Clinical evidence suggests that exposure history may influence therapeutic outcomes in EGFR-mutant NSCLC. Meta-analyses indicate that smoking history is associated with shorter progression-free and overall survival among patients treated with EGFR TKIs [28,29]. Mechanistically, cigarette smoke exposure has been shown to alter cellular metabolism and reduce EGFR-TKI sensitivity through pathways involving LKB1/AMPK signaling and metabolic reprogramming [30].

These observations suggest that environmental exposures may act as a biological conditioning layer that shapes signaling network organization during tumor evolution. Environmental and occupational exposures may therefore influence lung cancer biology by shaping the signaling context in which oncogenic driver mutations operate.

### 1.4. Rationale and Objectives of This Review

Despite significant therapeutic advances, clinical outcomes remain variable among patients with comparable genomic alterations. Recent evidence indicates that cancer biology is influenced not only by tumor-intrinsic mutations but also by interactions between environmental exposures, host physiology, and tumor microenvironmental dynamics [16].

In this context, the exposome represents a complementary dimension of precision oncology that captures the cumulative environmental and lifestyle factors that influence carcinogenesis and treatment response [31,32]. Advances in exposomics—including exposome-wide association studies and multi-omics integration—now enable systematic investigation of environmental influences on disease biology and therapeutic vulnerability [15,33].

The impact of environmental and workplace exposures on resistance to targeted therapies has not been fully explored in lung cancer research. New findings suggest that inhalational exposures like cigarette smoke, air pollution, and workplace chemicals may affect how tumors change, adapt, and respond to treatment [21,34].

While resistance to EGFR and ALK inhibitors has been extensively characterized at genetic and signaling levels, the contribution of environmental and occupational exposures to these processes remains insufficiently integrated into current frameworks.

This review addresses the gap by integrating mechanistic, epidemiologic, and clinical evidence linking the respiratory exposome to signaling plasticity, adaptive resistance mechanisms, and treatment outcome variability. Particular emphasis is placed on identifying exposure-associated signaling interfaces and clinically relevant endpoints that may influence the durability of EGFR- and ALK-targeted therapies.

Accordingly, we examine how major components of the respiratory exposome—including cigarette smoke, environmental tobacco smoke, cooking fumes, ambient air pollution, and occupational inhalational toxicants—may influence the effectiveness of EGFR- and ALK-targeted therapies in NSCLC, and discuss implications for patient stratification, rational combination strategies, and future clinical trial design.

While prior reviews have predominantly focused on genomic alterations and intracellular signaling mechanisms underlying resistance to EGFR and ALK inhibitors, the role of the environmental and occupational respiratory exposome as a modulator of adaptive resistance has not been systematically integrated into current frameworks. To address this gap, we propose a novel, multi-level conceptual and translational model linking chronic inhalational exposures with signaling plasticity, pharmacokinetic variability, and resistance evolution. By integrating mechanistic, epidemiologic, and translational evidence, this work provides a more comprehensive understanding of treatment heterogeneity in oncogene-driven NSCLC.

This narrative review is designed as a hypothesis-generating synthesis integrating mechanistic, translational, and clinical evidence on the role of the respiratory exposome in modulating adaptive resistance to EGFR- and ALK-targeted therapies in NSCLC.

To support this synthesis, a structured literature search was conducted in PubMed/MEDLINE, Web of Science, and Scopus (January 2020–February 2026), combining targeted therapy-related and exposure-related keywords. The search identified a total of 503 records, of which approximately 280 were screened after duplicate removal, and 95 studies were included based on relevance to mechanistic, epidemiologic, or clinical aspects of the exposome–resistance framework. Given the narrative nature of this review, studies were selected through iterative screening, prioritizing recent peer-reviewed evidence with mechanistic, epidemiologic, or clinical relevance. Findings were synthesized qualitatively to develop a biologically grounded conceptual framework.

The following sections integrate current knowledge on resistance mechanisms in EGFR- and ALK-driven NSCLC with emerging evidence on environmental and occupational respiratory exposures. In this framework, chronic inhalational exposures are conceptualized as dynamic modifiers of tumor signaling states that may influence adaptive resistance and therapeutic response.

### 1.5. Levels of Evidence Linking Respiratory Exposures to Targeted Therapy Resistance

Several studies have found links between respiratory exposures and resistance to targeted therapies in NSCLC [16,23]. For clarity, the evidence discussed in this review is interpreted within three categories: (i) established mechanisms supported by clinical or validated molecular data, (ii) emerging evidence derived from experimental and translational studies, and (iii) hypothesis-generating associations based primarily on mechanistic or epidemiological observations. This structured distinction is applied throughout the manuscript and summarized in Table 1 to facilitate interpretation of the exposome–resistance relationship.

Epidemiologic and experimental studies show that respiratory exposures, such as cigarette smoke, air pollution, and occupational inhalational toxicants, induce oxidative stress, epithelial injury, and persistent inflammatory signaling in lung tissue [35,41]. These changes create a pro-inflammatory and oxidative microenvironment that favors activation of oncogenic signaling pathways.

Mechanistic and phosphoproteomic studies show that targeted kinase inhibition can trigger adaptive remodeling of intracellular signaling networks, including activation of Src/FAK-associated modules and MET-mediated bypass pathways [23,30,42,43]. These findings illustrate the plasticity of receptor tyrosine kinase networks during EGFR- and ALK-targeted therapy.

Studies using single-cell sequencing and circulating tumor DNA demonstrate heterogeneous resistance trajectories that involve drug-tolerant persister states and adaptive transcriptional programs during targeted therapy [9,12].

Clinical evidence also suggests that a patient’s exposure history can affect treatment results. Studies and meta-analyses have found that people who have smoked respond less well and have shorter periods without disease progression when treated with EGFR TKIs [28,29].

Together, these complementary lines of evidence from exposure biology, signaling studies, evolutionary analyses, and clinical observations provide the empirical basis for the exposome-driven adaptive resistance framework discussed in the following sections. This framework extends existing models by incorporating environmental and occupational exposure dimensions into resistance biology.

## 2. Canonical Mechanisms of Resistance to EGFR-Targeted Therapies

### 2.1. On-Target Resistance Mutations

Resistance to EGFR TKIs often develops because of secondary changes in the EGFR kinase domain. In the past, the EGFR T790M mutation has made up about 50 to 60 percent of resistance cases to first-generation EGFR-TKIs [44].

Third-generation inhibitors such as osimertinib were developed to address T790M-related resistance and have significantly improved outcomes for patients with EGFR-mutant NSCLC [2]. Nevertheless, resistance to osimertinib still develops, often due to new EGFR mutations including C797S, which interfere with drug binding in the kinase domain [11].

Recent genomic studies show that resistance mutations often appear as combinations of several EGFR changes within the same tumor. These combined mutations can change the shape of the kinase binding pocket and make tumors less sensitive to different generations of inhibitors, which limits how long TKI therapy remains effective [2,45].

Studies using evolutionary and single-cell analysis suggest that resistant cell clones may already be present in small numbers before treatment begins. When EGFR is blocked, these clones proliferate and eventually become the dominant cell type in the tumor, illustrating how clonal selection drives resistance [9].

### 2.2. Bypass Pathway Activation

*In* addition to on-target mutations, activation of alternative signaling pathways represents a major mechanism of resistance to EGFR-targeted therapies. Amplification or activation of receptor tyrosine kinases, such as MET, HER2, and AXL, can restore downstream signaling through the PI3K/AKT and MAPK pathways despite EGFR inhibition [46].

MET amplification has been reported in approximately 5–20% of cases of EGFR-TKI resistance, with frequencies approaching ~20–25% in later-line resistance settings [2,3,47,48]. Similarly, HER2 amplification and AXL activation have been implicated in adaptive resistance and are frequently associated with increased cellular plasticity and EMT phenotypes [46].

From a systems biology perspective, bypass activation reflects the inherent redundancy and adaptability of receptor tyrosine kinase networks. Rather than functioning as isolated genomic events, these mechanisms emerge through dynamic signaling network rewiring, allowing tumor cells to maintain proliferative signaling under therapeutic pressure [49,50].

### 2.3. Phenotypic Plasticity and Microenvironmental Influences

Resistance to EGFR-targeted therapies can also involve non-genetic mechanisms related to phenotypic plasticity and tumor microenvironmental interactions. EMT represents a key adaptive resistance mechanism and is discussed in detail in Section 5.5.

At the same time, signals from the tumor microenvironment can keep survival pathways active even without EGFR activity. Inflammatory signaling pathways (see Section 5.4) contribute to microenvironmental support of resistance through cytokine-mediated survival signaling [51]. These adaptive cell states and influences from the microenvironment show that EGFR-TKI resistance is a complex, dynamic process involving changes in gene expression, signaling overlap, and interactions within the tumor ecosystem, not just genetic changes.

## 3. Resistance Biology in ALK-Rearranged NSCLC: Context for Exposure-Associated Modulation

### 3.1. ALK Resistance Overview

Resistance to ALK TKIs frequently arises due to secondary mutations within the ALK kinase domain. Mutations such as L1196M, G1202R, G1269A, C1156Y, and F1174L alter drug binding while preserving kinase activity [2,52].

Next-generation ALK inhibitors, such as alectinib, brigatinib, and lorlatinib, were designed to address these mutations, improve activity against resistant variants, and enhance central nervous system penetration [53]. However, resistance often develops through compound ALK mutations that accumulate under sequential therapeutic pressure and reduce sensitivity to multiple inhibitors [54].

These findings highlight the evolutionary nature of resistance in ALK-rearranged NSCLC, where clonal diversification during targeted therapy leads to varied resistance pathways [2].

### 3.2. Bypass Signaling

Besides kinase-domain mutations, activation of bypass signaling pathways is a key way that ALK-rearranged tumors develop resistance. Other receptor tyrosine kinases such as EGFR, MET, HER family receptors, and FGFR3 can reactivate MAPK and PI3K/AKT signaling even when ALK is effectively blocked [55,56].

These bypass mechanisms show how receptor tyrosine kinase signaling networks are both redundant and adaptable. Instead of acting as separate genomic events, these pathways develop through changes in signaling that help tumor cells keep growing even during treatment [2].

### 3.3. Additional Adaptive Mechanisms

Other factors that lead to resistance in ALK-rearranged NSCLC include crosstalk between receptor tyrosine kinases and changes driven by the tumor’s environment. Studies show that activating EGFR signaling can partly restore MAPK and PI3K pathway activity during ALK inhibition, which highlights how oncogene dependency in lung cancer can change depending on the situation [57]. This cross-activation can happen through ligand-driven receptor activation, changes in receptor movement, or loss of negative regulators like MIG6 [38].

Spatial and environmental factors can also affect how resistance develops. The central nervous system is a common place where ALK-rearranged NSCLC progresses, partly because early ALK inhibitors did not cross the blood–brain barrier well. While newer drugs like alectinib, brigatinib, and lorlatinib work better in the brain [2,53], microenvironmental differences between pulmonary and central nervous system (CNS) niches may still shape therapeutic response.

Overall, these findings show that resistance in oncogene-driven lung cancer develops in complex interactions between signaling plasticity and environmental context, not just from single genetic changes. This view suggests that factors like long-term environmental and workplace exposures may also affect how tumors adapt and respond to treatment. The following sections examine how components of the respiratory exposome may interact with resistance-associated signaling pathways in NSCLC.

## 4. The Occupational and Environmental Respiratory Exposome in Lung Cancer Biology

### 4.1. The Respiratory Exposome Framework

The respiratory exposome offers a framework for understanding how cumulative inhalational exposures affect lung cancer biology throughout life [15,16]. Although cigarette smoking has been the primary focus of lung cancer research, the respiratory environment is also influenced by a range of environmental and occupational toxicants, including ambient air pollution, environmental tobacco smoke, indoor combustion products, and workplace exposures such as diesel exhaust particles, crystalline silica, and metal aerosols [20,21,22].

Chronic exposure to inhaled toxicants induces biological responses involving oxidative and inflammatory signaling pathways, discussed in detail in Section 5.4, which contribute to tumor development and progression [58,59,60,61,62].

The respiratory exposome serves as a biological interface through which environmental and occupational exposures can affect lung cancer initiation, tumor evolution, and therapeutic response.

### 4.2. Cigarette Smoke as a Model of Exposure-Driven Signaling Rewiring

Cigarette smoke is the most extensively studied inhalational exposure in lung cancer and serves as a model for how chronic toxicant exposure alters oncogenic signaling networks [42,63]. Tobacco smoke contains thousands of chemicals that induce oxidative stress, DNA damage, and inflammatory signaling in airway epithelial cells [63].

Experimental studies show that nicotine and other smoke-derived compounds activate epidermal growth factor receptor (EGFR) signaling through nicotinic acetylcholine receptors, promoting proliferation and survival in lung epithelial cells [42,64]. Cigarette smoke also activates downstream pathways such as MAPK, NF-κB, and WNT/β-catenin, which contribute to tumor progression and inflammation in lung tissue [63,65].

Cigarette smoke exposure may influence therapeutic response in oncogene-driven lung cancer. Cheng et al. (2020) found that cigarette smoke extract reduces sensitivity to EGFR TKIs by suppressing LKB1 expression via CpG-island methylation, which enhances glycolysis and alters AMPK–mTOR signaling [30]. Tobacco-associated mutational signatures and co-occurring genomic alterations, such as TP53 mutations, have been linked to accelerated resistance development in EGFR-mutant lung adenocarcinoma [39].

Collectively, these observations suggest that chronic inhalational exposures may alter oncogenic signaling networks and potentially influence therapeutic vulnerability in lung cancer.

### 4.3. Occupational Inhalational Toxicants: Mechanistic Parallels and Distinctive Features

Occupational inhalational exposures are an important but often underrecognized part of the respiratory exposome [58,60]. Workers in industries such as mining, construction, metallurgy, and manufacturing may be exposed to toxicants like crystalline silica, diesel exhaust particles, welding fumes, and heavy metals. These exposures have been associated with relative risks for lung cancer of approximately 1.3 to 1.6 in occupational cohorts [58]. Rare but severe occupational malignancies, such as pleural malignant mesothelioma, further illustrate the long-term carcinogenic potential of inhalational exposures in occupational settings, particularly in association with asbestos exposure [66].

These exposures induce persistent epithelial injury and exposure-associated inflammatory and oxidative signaling responses (see Section 5.4), contributing to lung cancer development [58,67]. Mechanistic studies show that occupational toxicants activate signaling pathways involved in tumor progression, including NF-κB, MAPK, JAK/STAT, and aryl hydrocarbon receptor networks [19,59].

For example, hexavalent chromium exposure activates inflammatory cascades involving COX-2, VEGF, MAPK, and NF-κB signaling in lung tissue [59]. Crystalline silica similarly induces pulmonary inflammation by activating NF-κB signaling and the NLRP3 inflammasome, highlighting the role of innate immune signaling in exposure-related lung injury [37,68].

Because these pathways overlap with signaling networks involved in tumor progression and therapeutic resistance, occupational inhalational exposures may influence tumor evolution through persistent inflammatory and stress-response signaling within the lung microenvironment [59,67].

### 4.4. Environmental and Indoor Air Pollution

Ambient air pollution is a major environmental component of the respiratory exposome and a growing contributor to lung carcinogenesis [21,34,69]. Large cohort studies show that long-term exposure to fine particulate matter (PM2.5) increases lung cancer risk by about 10–20% for every 10 μg/m^3^ increase in exposure, particularly at levels exceeding current WHO guideline thresholds (>10 μg/m^3^ annual mean) [21,41,70]. Fine particulates and combustion-derived pollutants reach deep into lung tissue, causing oxidative stress, DNA damage, and chronic inflammation.

Experimental and epidemiologic evidence further shows that particulate pollutants activate oncogenic pathways, including PI3K/Akt, NF-κB, JAK/STAT, MAPK, and Wnt/β-catenin, thereby influencing proliferation and inflammatory responses in lung tissue [21,36,71]. Beyond carcinogenic initiation, air pollution may also promote tumor progression by stimulating macrophage recruitment and interleukin-1β-mediated inflammatory signaling in epithelial cells harboring oncogenic mutations such as EGFR or KRAS [35,72].

Indoor exposures further contribute to the respiratory exposome. Environmental tobacco smoke and cooking-related combustion products generate particulate matter and polycyclic aromatic hydrocarbons that can induce oxidative stress and inflammatory signaling in airway epithelial cells [73,74]. Genomic studies of lung cancer in never-smokers reveal molecular profiles enriched for EGFR alterations, suggesting interactions between environmental exposures and oncogenic signaling pathways [5,75].

### 4.5. Integrative Perspective: Chronic Exposures as Selective Pressures

Within the respiratory exposome framework, chronic inhalational exposures act as modifiers of the pulmonary signaling environment, influencing tumor development and progression [15,16]. These effects are mechanistically detailed in Section 5.4.

## 5. Exposome-Driven Signaling Rewiring: Molecular Interfaces

Environmental exposures may intersect with resistance pathways through mechanisms detailed in Section 5.4, potentially contributing to context-dependent reconfiguration of receptor tyrosine kinase networks, based primarily on experimental and mechanistic evidence [6,35]. Phosphoproteomic profiling of EGFR-TKI-resistant NSCLC models has demonstrated altered phosphorylation of more than 50 kinase substrates across the PI3K/AKT, MAPK, and focal adhesion signaling networks, illustrating extensive adaptive kinome remodeling under targeted-therapy pressure [23]. Functional studies identified FAK/Src-centered signaling modules as key nodes of adaptive resistance in osimertinib-resistant models [43]. Environmental exposures may contribute to this signaling plasticity by inducing inflammatory and oxidative stress pathways that enhance survival signaling in cells harboring oncogenic driver mutations [35].

### 5.1. Aberrant EGFR Phosphorylation and Ligand-Independent Activation

EGFR signaling typically begins with ligand binding, receptor dimerization, and kinase activation, which then activates MAPK and PI3K/AKT pathways [2]. Environmental exposures can induce ligand-independent EGFR phosphorylation, thereby sustaining oncogenic signaling in the absence of canonical receptor activation. Experimental studies show that cigarette smoke extract increases EGFR phosphorylation by approximately 1.5–2-fold and reduces EGFR-TKI sensitivity by 30–50% in vitro [30,43].

Evolutionary analyses suggest that resistant cell subpopulations may exist before therapy and expand under EGFR-TKI selective pressure [9,12].

### 5.2. Src and FAK Activation as Adaptive Kinome Nodes

Adaptive resistance to targeted therapies often involves kinome reprogramming rather than single-gene changes, reflecting dynamic signaling network reorganization under therapeutic pressure [6,40,76]. In these networks, Src family kinases and FAK serve as central hubs, integrating receptor tyrosine kinase signaling with cytoskeletal remodeling, cell survival, and migration. This sustains MAPK and PI3K/AKT signaling despite EGFR inhibition [77].

Functional studies identify Src–FAK signaling modules as critical adaptive resistance nodes. Experimental models show a two- to four-fold increase in Src phosphorylation during EGFR-TKI resistance [43].

Adaptive signaling modules involving AXL and SRC also contribute to resistance in EGFR-mutant lung cancer models, underscoring the role of kinome-level signaling plasticity in reducing oncogene dependency [77].

Overall, these findings support a model where adaptive kinome nodes, especially Src, FAK, and AXL-centered modules, act as key mediators of non-genetic resistance to EGFR-targeted therapies.

### 5.3. c-MET-Mediated Bypass Signaling

Bypass receptor tyrosine kinase activation is a key resistance mechanism to EGFR-targeted therapies. MET amplification and activation are established drivers of resistance in EGFR-mutant NSCLC [47,48]. MET signaling can restore PI3K/AKT and MAPK pathway activity despite EGFR inhibition, supporting tumor cell survival and proliferation [44]. Experimental studies show that disrupted receptor trafficking and degradation can stabilize both EGFR and MET signaling, reinforcing oncogenic networks [78]. New therapies targeting EGFR and MET degradation show promise in overcoming resistance in EGFR-TKI-resistant lung cancer models [79]. These findings emphasize the central role of MET-driven bypass signaling in adaptive resistance.

### 5.4. Redox and Inflammatory Pathway Integration

Experimental and mechanistic studies indicate that chronic inhalational exposures are associated with increased oxidative stress and inflammatory signaling in lung tissue [41,80]. In experimental models, particulate matter exposure has been shown to increase reactive oxygen species in lung epithelial cells by approximately two- to three-fold, amplifying inflammatory pathways in tumor progression [41].

Inflammatory cytokines activate signaling networks such as STAT3, NF-κB, and PI3K/AKT, which maintain survival signaling independently of EGFR or ALK activation [81,82]. Environmental particulate exposure promotes tumor development by recruiting macrophages and activating interleukin-1β-mediated inflammatory signaling in epithelial cells with oncogenic driver mutations [35].

Occupational toxicants such as hexavalent chromium and other industrial aerosols similarly induce inflammatory cascades involving NF-κB and MAPK signaling, reinforcing a persistent inflammatory microenvironment that may support tumor progression and adaptive signaling [59].

### 5.5. EMT Induction and Phenotypic Plasticity

Phenotypic plasticity represents a central determinant of resistance evolution in oncogene-driven lung cancer. EMT reduces dependence on EGFR signaling while increasing invasive and stem-like cellular properties and has been reported in approximately 5–15% of cases of EGFR-TKI resistance in clinical series [83,84]. Contemporary integrative analyses indicate that EMT is accompanied by coordinated activation of bypass receptor tyrosine kinases such as AXL and MET, which reorganize intracellular signaling hierarchies and attenuate strict oncogene dependency [85]. Mechanistically, EMT-associated resistance states exhibit increased reliance on alternative kinase signaling nodes and inflammatory transcriptional programs that support survival under targeted-therapy pressure [86]. Clinical syntheses further indicate that EMT-positive tumors exhibit reduced duration of response to EGFR-TKIs, often below 8–10 months and frequently require combinatorial therapeutic strategies rather than sequential monotherapy [6,7]. Chronic inflammatory and oxidative stress induced by environmental exposures may further facilitate EMT-like transitions and promote signaling heterogeneity within tumor cell populations [75].

### 5.6. Epigenetic and Transcriptional Reprogramming

Drug-tolerant persister states are reversible adaptive phenotypes that result from transcriptional and epigenetic remodeling rather than stable genetic mutations [6,40]. Single-cell and multi-omic analyses of EGFR-mutant NSCLC reveal significant transcriptional heterogeneity during resistance evolution, indicating diverse adaptive programs that precede stable genomic resistance [9]. These persister states may serve as reservoirs for more durable resistance mechanisms under ongoing targeted therapy [40].

Non-genetic resistance programs are often strengthened by epigenetic–metabolic coupling. Changes in histone modifications, such as methylation and acetylation, can alter chromatin accessibility and sustain pro-survival pathways in EGFR-TKI-resistant cells [23,87]. Metabolic changes may further stabilize drug-tolerant states by modifying chromatin structure and transcriptional programs involved in stress responses and proliferation [23].

RNA epitranscriptomic regulation adds another layer of control over transcript stability and translation efficiency in EGFR-targeted therapy models [23,88]. In exposure-conditioned settings, chronic oxidative stress and inflammatory cues from environmental toxicants may promote epigenetic plasticity before therapy [41,80]. These processes can alter DNA methylation and chromatin organization, supporting the emergence of drug-tolerant cell states during targeted therapy [59].

Functional kinome studies indicate that kinase network activity contributes to the maintenance of drug-tolerant states, reinforcing adaptive resistance in EGFR-mutant NSCLC [40,88].

**Figure 1:**
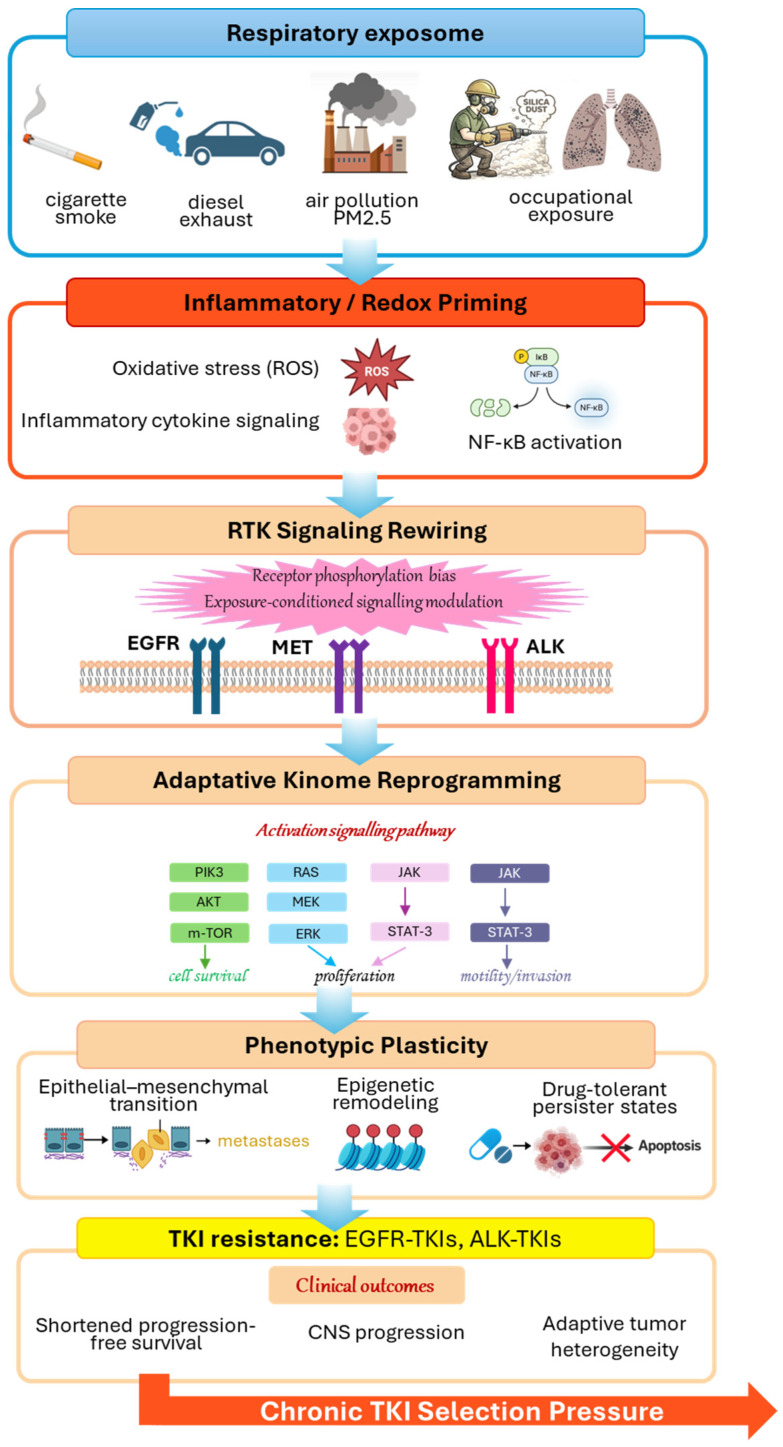
Exposome-driven signaling rewiring in EGFR- and ALK-targeted NSCLC. The schematic illustrates molecular signaling interfaces through which environmental exposures induce oxidative stress, inflammatory activation, and adaptive kinome remodeling, leading to receptor tyrosine kinase signaling reconfiguration. These exposure-associated processes promote phenotypic plasticity, including epithelial–mesenchymal transition and epigenetic remodeling, ultimately contributing to resistance to EGFR and ALK tyrosine kinase inhibitors and to heterogeneous clinical outcomes such as shortened progression-free survival. Created with BioRender.com.

As illustrated in Figure 1, exposure-associated signaling rewiring integrates oxidative stress, inflammatory activation, and adaptive kinome remodeling within the broader process of resistance evolution.

## 6. Pharmacokinetic and Metabolic Modulation by Environmental and Occupational Exposures

Environmental exposures—including cigarette smoke and combustion-derived pollutants—may influence targeted therapy efficacy through pharmacokinetic mechanisms, including induction of xenobiotic-metabolizing enzymes such as CYP3A and modulation of drug transporter activity [3,89,90]. Chronic inhalational exposures may alter drug metabolism, transporter activity, and tissue distribution, changing intratumoral exposure to TKIs even in genomically sensitive tumors [3,89].

### 6.1. Modulation of CYP450-Mediated Drug Metabolism

EGFR and ALK inhibitors are mainly metabolized by hepatic cytochrome P450 enzymes, especially CYP3A4 and CYP3A5 [2]. CYP3A-mediated metabolism accounts for over 70–80% of systemic clearance for several EGFR tyrosine kinase inhibitors, including osimertinib and erlotinib [3].

Environmental and occupational exposures may influence drug metabolism through modulation of xenobiotic-metabolizing pathways, including activation of nuclear receptor signaling (e.g., AhR and PXR) and downstream CYP1A- and CYP3A-dependent systems [82,90,91]. CYP1A enzymes (CYP1A1/1A2) are key mediators of exposure-driven metabolism, being induced via aryl hydrocarbon receptor activation by polycyclic aromatic hydrocarbons and other inhalational toxicants, with experimental systems showing ~1.4–1.8-fold increases in expression and up to 170–180% increases in enzymatic activity [90,92].

In contrast, CYP3A enzymes are primarily responsible for the metabolism and pharmacokinetics of clinically used drugs, including EGFR and ALK tyrosine kinase inhibitors, and represent a major determinant of systemic drug exposure [3,93,94,95].

Clinical data demonstrates substantial inter-individual variability in osimertinib exposure (up to three-fold), with inflammatory signaling (e.g., interleukin-6 levels) also associated with increased systemic drug exposure, highlighting the sensitivity of targeted therapy pharmacokinetics to CYP3A modulation [3,93,96].

Overall, CYP1A pathways reflect exposure-driven metabolic activation, whereas CYP3A pathways determine clinically relevant drug metabolism, suggesting a potential link between environmental exposures and variability in targeted therapy pharmacokinetics.

### 6.2. Transporter Regulation and Intracellular Drug Accumulation

Beyond hepatic metabolism, drug transporters are critical in determining intracellular drug concentrations. Efflux transporters such as P-glycoprotein (ABCB1) and breast cancer resistance protein (ABCG2) control the cellular accumulation of many targeted therapies and influence drug penetration across physiological barriers, including the blood–brain barrier.

Recent clinical evidence shows that germline variation in ABCB1 and ABCG2 may affect intracranial disease control in patients treated with osimertinib, highlighting the role of transporter-mediated pharmacokinetics in therapeutic variability [97]. Experimental models also show that inflammatory signaling and oxidative stress, common after chronic inhalational exposure, can regulate transporter expression through NF-κB and AhR-dependent pathways [98].

Upregulation of efflux transporters in tumor cells or barrier tissues may reduce intracellular TKI concentrations, even if plasma drug levels are adequate.

### 6.3. Tissue Distribution and CNS Pharmacokinetics

Changes in tissue drug distribution may further link environmental exposures to therapeutic variability. Chronic inflammation from inhalational toxicants can alter vascular permeability, extracellular matrix structure, and interstitial pressure, affecting drug diffusion within tumor tissue. Tumor-intrinsic signaling programs may also contribute to CNS progression during targeted therapy. Experimental studies show that RhoA-dependent signaling promotes brain metastatic outgrowth and reduces sensitivity to osimertinib in EGFR-mutant NSCLC models [99].

The central nervous system is a key pharmacokinetic compartment in EGFR- and ALK-driven NSCLC. Central nervous system progression remains a frequent clinical pattern of treatment failure during EGFR- and ALK-targeted therapy, even in patients who initially achieve systemic disease control [2,53]. Although next-generation inhibitors such as osimertinib and lorlatinib have improved blood–brain barrier penetration [2], transporter-mediated efflux and microenvironmental factors still affect intracranial drug exposure. Emerging evidence indicates that inflammatory signaling and vascular remodeling may further alter blood–brain barrier transport, contributing to variable CNS progression during targeted therapy [100,101].

### 6.4. Integrative Perspective

In summary, environmental and occupational exposures may affect targeted therapy efficacy through pharmacokinetic mechanisms as well as signaling rewiring. Changes in CYP450 metabolism, drug transporter expression, and tissue distribution can alter intratumoral exposure to TKIs and impact therapeutic durability [2,97]. Within an exposome-informed framework, pharmacokinetic modulation represents an additional mechanism through which environmental exposures may contribute to variability in targeted therapy response.

## 7. Clinical Correlates: Exposure-Associated Variability in Treatment Outcomes

Mechanistic evidence summarized in previous sections indicates that respiratory exposures may influence targeted therapy efficacy through signaling and pharmacokinetic modulation [16,35]. The clinical literature, although still evolving, provides supporting evidence for exposure-associated variability in response rates, progression-free survival, and resistance trajectories in EGFR- and ALK-driven NSCLC [28,29].

Exposure history may represent a biologically plausible modifier of therapeutic vulnerability, although current evidence is largely indirect [16,31].

### 7.1. Smoking Status and EGFR-TKI Outcomes

Clinically, smoking represents the most consistently documented exposure associated with variability in EGFR-TKI outcomes in EGFR-mutant NSCLC. Multiple retrospective cohort studies and meta-analyses demonstrate that smoking history is associated with shorter progression-free survival (PFS) and reduced objective response rates among patients treated with EGFR TKIs [28,29].

Pooled analyses report significantly longer progression-free survival in never-smokers compared with smokers (HR 0.73, 95% CI 0.60–0.88) [28], corresponding to an absolute difference of approximately 4–6 months in median PFS (12–16 vs. 8–10 months). Consistent with these findings, clinical cohorts demonstrate reduced treatment durability in smokers receiving EGFR-targeted therapy [2,7].

In experimental models, cigarette smoke exposure induces exposure-associated signaling adaptations (as detailed in Section 5.4) that may reduce tumor dependency on EGFR signaling [24,30]. Smoking-associated tumors frequently harbor co-occurring genomic alterations and mutational signatures that may accelerate resistance evolution during targeted therapy [64].

These observations are supported by experimental data showing that smoke exposure promotes EGFR phosphorylation, Src activation, and metabolic rewiring, all of which may diminish EGFR-TKI sensitivity [30]. Taken together, these observations indicate that smoking may weaken oncogene addiction and reduce the durability of EGFR-targeted therapy in susceptible patients.

### 7.2. Environmental and Occupational Modulation of ALK-TKI Efficacy

Evidence linking environmental or occupational exposures with treatment outcomes in ALK-rearranged NSCLC remains limited compared with EGFR-mutant disease. However, mechanistic studies suggest that exposure-related inflammatory signaling and tumor microenvironment alterations may influence the efficacy of ALK-targeted therapies.

Resistance to ALK inhibitors frequently involves activation of bypass signaling pathways—including EGFR, MET, and HER family receptors—which restore downstream PI3K/AKT and MAPK signaling despite ALK inhibition, with bypass pathway activation reported in approximately 20–30% of resistance cases [2,55]. Occupational inhalational toxicants therefore provide mechanistic plausibility for exposure-related modulation of targeted therapy response [35]. Environmental exposures capable of inducing chronic inflammatory signaling—such as tobacco smoke or particulate pollution—may indirectly influence ALK-TKI responsiveness through shared adaptive signaling pathways, including MAPK and PI3K/AKT network activation [4,20].

Central nervous system progression also represents an important determinant of treatment duration in ALK-positive NSCLC and occurs in up to 30–50% of patients treated with earlier-generation ALK inhibitors, reflecting limited drug penetration across the blood–brain barrier [2,4,53]. Although occupational exposure histories are rarely reported in oncologic cohorts, chronic inhalation of industrial toxicants may contribute to persistent inflammatory signaling and potentially modify therapeutic responses [16].

Thus, smoking represents a clinically validated exposure associated with treatment variability, whereas the effects of other respiratory exposures should be considered biologically plausible but not yet clinically established.

### 7.3. Population-Specific Patterns

Exposure rates differ by region and demographic group, resulting in variability in targeted therapy outcomes across studies. For instance, lung cancers in never-smokers, particularly in East Asian populations, exhibit higher rates and distinct patterns of EGFR mutations compared to smoking-related cancers [5]. Environmental factors such as indoor combustion, cooking fumes, and air pollution may contribute to these regional differences in lung cancer biology [36,73]. These variations complicate direct comparisons between clinical trials from different regions. Studies with predominantly never-smokers may report higher response rates to EGFR-targeted therapies than those with more smokers. Incorporating environmental and occupational exposure data into clinical trial design could clarify inter-study differences and enhance precision oncology by accounting for exposure-related biological variation.

### 7.4. Exposure History as a Clinical Biomarker

Cumulative epidemiologic and translational evidence suggests that respiratory exposure history may be associated with variability in therapeutic response in oncogene-driven NSCLC. Growing epidemiologic and translational studies indicate that chronic inhalational exposures, including cigarette smoke, environmental pollution, and indoor combustion products may be associated with remodeling of tumor signaling networks and influence the durability of response to EGFR and ALK TKIs [15,16].

Among these exposures, cigarette smoking remains the most consistently documented factor associated with reduced EGFR-TKI efficacy. Meta-analyses and cohort studies demonstrate that smoking history correlates with shorter progression-free survival and diminished treatment response in EGFR-mutant NSCLC [28,29]. Mechanistically, tobacco smoke induces exposure-associated signaling adaptations (see Section 5.4), which may weaken strict oncogene dependency and facilitate adaptive signaling diversification [30,42].

Other components of the respiratory exposome may similarly contribute to signaling network remodeling within lung tissue [35,36,58,59,73,79]. However, it is critical to distinguish between clinically validated observations and hypothesis-generating associations. While smoking-related effects are supported by consistent clinical outcome data demonstrating reduced EGFR-TKI efficacy [28,29], the impact of other environmental and occupational exposures remains largely inferred from mechanistic and epidemiologic studies [34,36,58,59,73,79] and has not yet been validated in prospective clinical settings.

Taken together, these exposures may create biologically distinct tumor states characterized by increased inflammation, greater bypass signaling, and enhanced phenotypic plasticity under targeted therapy. Table 1 summarizes the main clinical and mechanistic links between respiratory exposures and variability in targeted therapy outcomes.

Evidence levels reflect the main type of supporting data available, from epidemiologic associations and clinical outcome studies to mechanistic and experimental investigations. Prospective exposure-stratified oncology trials are limited, especially for occupational inhalational toxicants [16,31]. Most clinical evidence comes from EGFR-mutant NSCLC, but shared adaptive signaling nodes suggest potential relevance for ALK-rearranged disease [6,23].

## 8. Translational Implications

Integrating occupational and environmental exposures into resistance biology expands our understanding of oncogene-driven lung cancer. EGFR- and ALK-driven tumors evolve within environmentally conditioned tumor states, influenced by exposure-conditioned signaling states (see Section 5) that modulate responses to targeted therapy [15,16,17]. Experimental and translational evidence suggests that chronic inhalational exposures may modulate signaling dynamics and adaptive capacity during therapy [16,35]. Translationally, these factors indicate that therapeutic responses should be assessed within a framework that includes tumor genomics, host physiology, and exposure history [31].

### 8.1. Reframing Oncogene Addiction Under Environmental Pressure

The concept of oncogene addiction traditionally assumes dominant reliance on a single oncogenic signaling driver. Increasing evidence indicates that receptor tyrosine kinase signaling networks operate within adaptive signaling systems that can reorganize under environmental and therapeutic pressures [2,6].

Chronic inhalational exposures may create signaling environments where oncogene dependency is more context-dependent. In these cases, inflammatory signaling and kinase network plasticity can enable bypass pathway activation during targeted therapy, reducing the durability of oncogene-directed inhibition [24,30,35,42].

### 8.2. Exposure-Informed Combination Hypotheses

Mechanistic insights into resistance evolution support combination therapy strategies that target adaptive signaling circuits in oncogene-driven lung cancer. Activation of bypass receptor tyrosine kinases, such as MET, HER family receptors, and AXL, is a common resistance mechanism to EGFR-targeted therapies and can restore PI3K/AKT and MAPK signaling despite EGFR inhibition [46,47,48].

Adaptive Src/FAK signaling modules (Section 5.2) represent key kinome-level resistance nodes that may be therapeutically targetable in exposure-associated context.

These findings support combination strategies that target both the primary oncogenic driver and adaptive signaling nodes. For instance, dual inhibition of EGFR and MET has shown clinical activity in MET-dependent resistance [47,48], while targeting kinome components such as Src or FAK may suppress compensatory survival signaling during therapy [6,77].

Within an exposome-informed framework, exposure history may serve as a stratification variable, identifying tumors with inflammatory or kinase-biased signaling states that may derive greater benefit from such combinatorial therapeutic approaches [16,23,35].

### 8.3. Precision Oncology Beyond Genomics

Current precision oncology models focus on tumor genomic profiling, but genomic status alone may not fully predict treatment durability [2,6]. A broader framework that includes host factors and cumulative environmental exposures may better explain why tumors with similar oncogenic drivers follow divergent resistance trajectories during therapy [23,31].

Cumulative environmental and occupational exposure history adds an important dimension to the biological context that can influence signaling plasticity and treatment response [15,16]. Recent exposome-oriented studies highlight the value of combining environmental determinants with molecular profiling to better understand individual differences in cancer biology and outcomes [32,33]. Integrating these factors may help explain why tumors with similar oncogenic drivers can still follow different resistance trajectories during therapy.

Exposure-informed stratification may help identify patients at higher risk of rapid resistance, supporting intensified monitoring or early therapeutic intervention [16,31].

### 8.4. Clinical Trial Design Incorporating Exposome Stratification

Most clinical trials evaluating EGFR and ALK TKIs stratify patients primarily according to tumor genomic alterations and clinical characteristics. Emerging evidence suggests that environmental and occupational exposures may represent additional determinants of therapeutic variability that are rarely captured in oncology trial design [16,31].

Including exposure-related variables in prospective clinical studies may improve interpretation of varied treatment outcomes. Quantitative measures of tobacco exposure, such as cumulative pack-years, offer more informative stratification than binary smoking status, especially given the link between smoking history and reduced EGFR-TKI efficacy [28,29]. Similarly, documenting occupational inhalational exposures or regional air pollution indices can help contextualize inflammatory and signaling states that affect tumor behavior [35,59].

While prospective validation is limited, integrating exposure metrics into clinical trial design is a useful step toward a more environmentally contextualized precision oncology framework.

### 8.5. Implications for Exposure-Stratified Precision Oncology

From a clinical perspective, integrating structured exposure assessment into routine oncologic evaluation may refine risk stratification beyond tumor genomics alone [16,31]. Exposure history may represent a practical stratification variable that complements tumor genomic profiling. Environmental and occupational exposures can influence tumor biology through inflammatory signaling, oxidative stress, and modulation of oncogenic pathways relevant to therapeutic response [15,23,35]. Consistent with this framework, smoking burden in EGFR-mutant NSCLC has been associated with shorter progression-free survival and reduced response to EGFR TKIs [28,29].

Incorporating exposure metrics, such as cumulative smoking burden, occupational inhalational exposure history, and inflammatory biomarker profiling, may enable more individualized monitoring and help interpret varied outcomes in targeted therapy trials [29,94]. Translationally, integrating exposure-associated biological signals, such as inflammatory cytokine profiles or phospho-kinome signatures, may clarify whether exposure-driven signaling changes contribute to variability in therapy durability [23,35].

**Figure 2:**
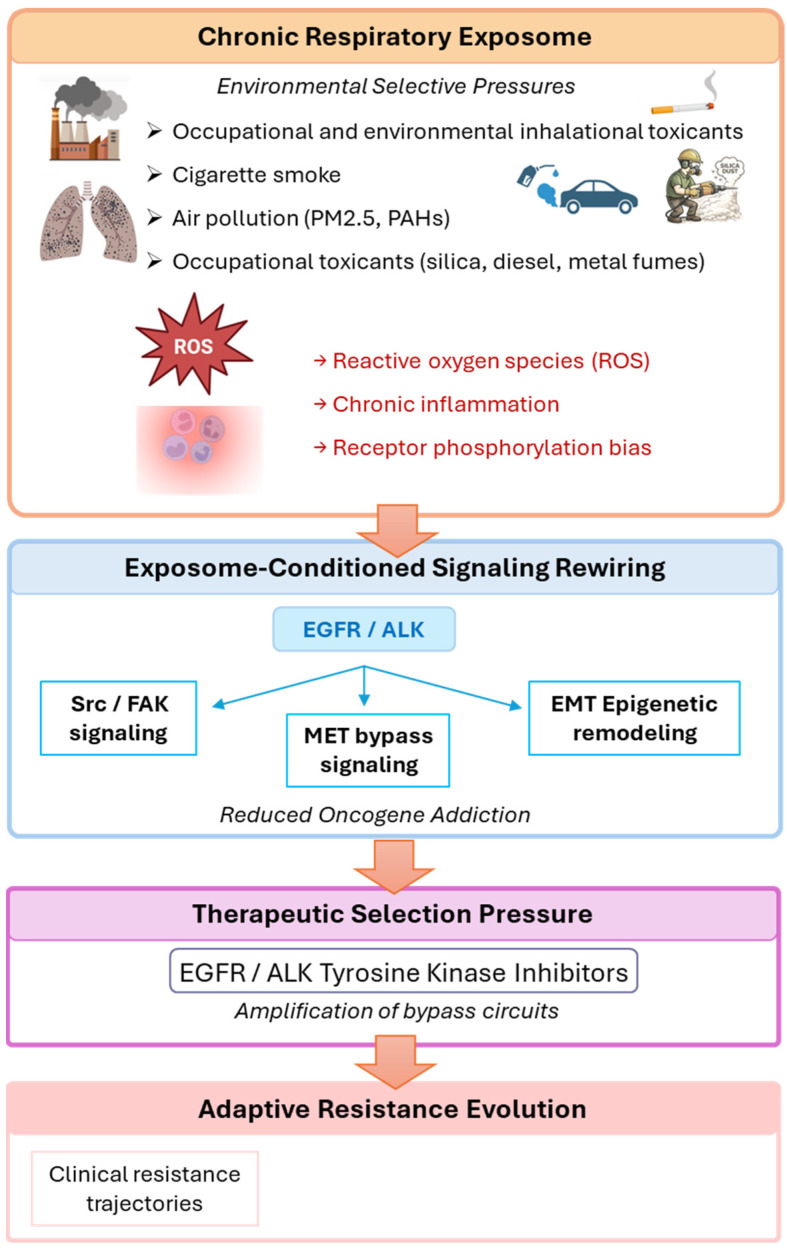
Evolutionary and temporal framework of exposome-driven adaptive resistance in EGFR- and ALK-targeted NSCLC. The schematic illustrates how chronic respiratory exposures act as persistent environmental selective pressures that shape tumor signaling states before and during targeted therapy. These exposure-conditioned states promote adaptive pathway activation, including Src/FAK signaling, MET bypass signaling, epithelial–mesenchymal transition, and epigenetic remodeling, thereby facilitating resistance evolution under the selective pressure of EGFR and ALK tyrosine kinase inhibitors. The model emphasizes the temporal dynamics of resistance development and resulting heterogeneous clinical trajectories, including shortened progression-free survival and central nervous system progression. Created with BioRender.com.

Figure 1 focuses on molecular signaling mechanisms, whereas Figure 2 illustrates the temporal and evolutionary integration of these processes within an exposome-driven framework.

At this stage, it is important to distinguish between established findings, biologically plausible mechanisms, and unproven areas. Smoking history is consistently linked to shorter progression-free survival in patients receiving EGFR tyrosine kinase inhibitors. Resistance mechanisms such as MET activation, Src/FAK signaling, and EMT are well recognized [28,29,48,77]. In contrast, while it is biologically plausible that environmental and occupational exposures influence signaling states and the durability of targeted therapy, this hypothesis has not been adequately tested in prospective exposure-stratified oncology cohorts.

## 9. Conceptual Model: Exposome-Driven Adaptive Resistance Framework

For clarity, evidence discussed in this section includes clinically validated mechanisms, preclinical/mechanistic findings, and hypothesis-generating associations. Resistance to EGFR- and ALK-targeted therapies is increasingly viewed as a multi-layered process involving interactions between tumor genomics, signaling plasticity, and environmental influences. Chronic respiratory exposures, including cigarette smoke and particulate pollution, may influence receptor tyrosine kinase signaling architecture through mechanisms detailed in Section 5.4 [20,35]. In this context, environmental exposures can be conceptualized as potential modifiers of tumor signaling states rather than isolated risk factors. However, this framework is biologically plausible and supported by convergent experimental and indirect clinical evidence, but it has not yet been validated in prospective exposure-stratified oncology cohorts.

The proposed framework is supported by exposure-associated signaling mechanisms (Section 5.4) that influence oncogenic pathways in lung tissue [35,41]. Phosphoproteomic analyses reveal adaptive kinome remodeling involving Src- and FAK-associated signaling networks during EGFR-TKI resistance [23,43]. Complementary single-cell studies demonstrate substantial transcriptional heterogeneity during resistance evolution in EGFR-mutant NSCLC [9,12].

Clinical data consistently indicate that smoking history is associated with shorter progression-free survival in patients receiving EGFR-targeted therapies (see Section 7.1) [28,29,102].

This integrative model is informed by quantitative evidence from molecular signaling studies, phosphoproteomic analyses, single-cell sequencing, and clinical outcome datasets, rather than representing a purely conceptual model.

Importantly, while smoking-associated clinical effects are well supported, the influence of other environmental and occupational exposures remains primarily supported by mechanistic and epidemiological evidence and should be interpreted with caution.

Within this perspective, resistance evolution reflects the interaction of four interrelated domains: environmental selective pressures, signaling network reconfiguration, resistance evolution dynamics, and therapeutic interception points.

Exposure-associated signaling alterations may accelerate resistance evolution, although the magnitude of this effect requires prospective validation in exposure-stratified clinical cohorts [2,6].

### 9.1. Environmental and Occupational Selective Pressures

Chronic inhalational exposures create a persistent biological environment characterized by epithelial injury and signaling alterations (see Section 5.4) [35,59,77]. Phosphoproteomic and signaling studies show that chronic cigarette smoke exposure remodels oncogenic signaling networks, activating Src-associated pathways, inflammatory cascades, and metabolic adaptations that can influence cellular survival and therapeutic response [30,80]. Smoking-related lung carcinogenesis is also linked to sustained NF-κB pathway activation, inflammatory cytokine production, and transcriptional programs associated with EMT and stem-like behavior [80].

Environmental exposures may also act as selective pressures at earlier stages of tumor evolution. In a landmark study, Hill et al. (2023) demonstrated that air pollution promotes tumor growth by acting on cells with pre-existing EGFR or KRAS alterations, favoring the expansion of oncogene-primed epithelial populations [35]. Similarly, Diaz-Gay et al. (2025) found that lung cancers in never-smokers from highly polluted environments have distinct mutational signatures and genomic features, indicating environmentally driven evolutionary changes [5]. These findings suggest that tumors may develop within an exposure-conditioned signaling ecosystem, in which oxidative stress, inflammatory signaling, and receptor phosphorylation biases exist before targeted therapy begins [5,35].

### 9.2. Signaling Network Reconfiguration

In exposure-conditioned environments, receptor tyrosine kinase signaling networks may be reconfigured, reducing strict oncogene dependency. Adaptive Src/FAK signaling modules (Section 5.2) illustrate how kinome plasticity supports adaptive resistance within this framework [77].

Kinome plasticity under targeted therapy reflects dynamic signaling reprogramming, involving chromatin remodeling, transcriptional adaptation, and shifts in pathway hierarchy, whereby cancer cells adjust kinase activity under therapeutic pressure [6,76]. These adaptive changes support the persistence of drug-tolerant cell states and the evolution of acquired resistance in oncogene-driven lung cancers [40].

This reconfiguration affects not only receptor activation but also downstream pathway bias and phenotypic plasticity. MET-driven bypass signaling is a common adaptive escape mechanism, with MET amplification and sustained signaling central to resistance across multiple TKI settings [8,48]. EMT (Section 5.5), together with transcriptional plasticity and epigenetic remodeling, contributes to reduced oncogene dependency within exposure-conditioned tumor states [9,76,85].

Conceptually, tumors exist along a continuum of driver reliance shaped by chronic exposure intensity and signaling redundancy, rather than in a binary sensitive or resistant state [9,49,76].

### 9.3. Resistance Evolution Dynamics

When targeted therapy begins, clonal selection occurs within this pre-existing biased signaling landscape. Single-cell genomic and transcriptomic analyses of EGFR-mutant NSCLC reveal significant intratumoral heterogeneity during resistance evolution, with multiple adaptive trajectories emerging under therapeutic pressure [9].

Rather than arising exclusively from de novo mutations during treatment, resistant states may originate from pre-existing minor subpopulations, drug-tolerant persister cells, or exposure-influenced signaling programs that are preferentially selected during therapy. Single-cell analyses of osimertinib-resistant tumors show that multiple driver alterations may coexist within individual cancer cells, illustrating the complexity of resistance architectures [12].

Longitudinal studies using circulating tumor DNA (ctDNA) show dynamic acquisition of resistance alterations during EGFR-targeted therapy, including secondary EGFR mutations and activation of bypass signaling pathways [12,13]. Reviews of resistance in EGFR- and ALK-driven NSCLC support a model where treatment-specific selective pressures promote both on-target and off-target escape mechanisms, such as bypass signaling and phenotypic transformation [2,6].

Drug-tolerant persister biology adds another layer of complexity. Experimental and single-cell studies indicate that persister cells survive therapy through reversible non-genetic adaptations involving metabolic rewiring, epigenetic remodeling, stress-response activation, and transcriptional plasticity, serving as reservoirs from which more stable resistant populations may emerge [10,11]. In exposure-conditioned contexts where inflammatory and oxidative pathways are already active, these adaptive trajectories may be further accelerated.

### 9.4. Therapeutic Interception Points

The exposome-based adaptive resistance model identifies several potential therapeutic targets. First, pre-therapy exposure assessment may refine risk stratification beyond tumor genomics [16,31]. The history of smoking burden, occupational inhalational exposure, and environmental pollution may identify patients more likely to exhibit adaptive signaling redundancy or reduced oncogene dependence within exposure-conditioned tumor ecosystems [15,28].

Second, early combination strategies targeting adaptive signaling nodes may be rational in exposure-conditioned tumors. For example, combined MET and EGFR inhibition has shown clinical promise in patients with acquired MET-dependent resistance, with meta-analytic data supporting meaningful response rates in selected NSCLC populations [103]. Preclinical and translational studies identify focal adhesion kinase (FAK) and Src family kinases as important adaptive signaling hubs contributing to resistance-associated signaling plasticity. FAK-driven resistance has been linked to integrin signaling and osimertinib tolerance [104], while the AXL/CDCP1/SRC signaling axis has been implicated in acquired resistance states that may be partially reversible with Src inhibition [77].

Third, pharmacokinetic monitoring may become more relevant in exposure-heavy patients if chronic environmental or occupational toxicants influence CYP activity, transporter expression, or tissue drug distribution [2,93,105]. Fourth, modulation of inflammatory signaling pathways may represent an adjunctive strategy in tumors supported by NF-κB-, JAK/STAT-, or cytokine-driven microenvironments [83]. Finally, epigenetic intervention aimed at preventing stabilization of drug-tolerant persister states may offer a strategy to limit the transition from transient tolerance to stable resistance phenotypes [11,106].

Taken together, these mechanisms illustrate how exposure history, signaling plasticity, and therapeutic selection pressures interact to influence resistance trajectories in oncogene-driven lung cancer. The integrated adaptive resistance architecture from these interacting layers of environmental pressure, signaling reconfiguration, and therapeutic selection is conceptually synthesized in Figure 2.

Several limitations should be acknowledged. Much of the evidence linking respiratory exposures to resistance to targeted therapies remains indirect, deriving primarily from mechanistic, preclinical, and exposure-associated epidemiologic studies rather than prospective oncology cohorts. In addition, occupational exposure variables are rarely captured systematically in clinical trials evaluating EGFR and ALK inhibitors. Consequently, the exposome-driven resistance framework should be considered biologically plausible, supported by convergent evidence, but not yet prospectively validated. Addressing these limitations will require prospective exposure-annotated studies integrating detailed exposure assessment, molecular profiling, longitudinal monitoring of resistance evolution, and treatment outcome data.

## 10. Future Directions and Research Priorities

The evidence reviewed above suggests that respiratory exposures may represent an underrecognized modifier of targeted therapy response [16,35], highlighting several important priorities for future translational and clinical research. Although mechanistic studies and retrospective clinical analyses increasingly suggest that respiratory exposures may influence the durability of targeted therapies, prospective validation remains limited. Addressing this gap will require multidisciplinary research integrating oncology, molecular biology, pharmacology, and occupational health sciences. The following research priorities may help translate the exposome-driven resistance framework into clinical research.

### 10.1. Prospective Exposure-Stratified Clinical Trials

Current clinical trials evaluating EGFR and ALK TKIs rarely incorporate detailed exposure metrics beyond basic smoking status. Accumulating clinical evidence indicates that smoking burden is associated with reduced efficacy of EGFR-TKIs and shorter progression-free survival in EGFR-mutant NSCLC [28,29]. Future prospective trials could benefit from systematically capturing quantitative indicators of respiratory exposure, including cumulative smoking burden, occupational inhalational exposure history, and environmental pollution indices. Integrating exposure variables into trial design may enable stratified analyses to evaluate whether exposure-heavy patient subgroups derive differential benefit from combination therapeutic strategies targeting adaptive signaling nodes, such as the MET or Src pathways [47,48]. Such studies would provide critical evidence regarding whether exposure-informed therapeutic adaptation can improve treatment durability and survival outcomes.

### 10.2. Multi-Omics Integration

Multi-omic investigations combining transcriptomics, epigenomics, proteomics, and metabolomics may help clarify how chronic inhalational exposures reshape tumor biology and resistance evolution [49,76]. Single-cell analyses have demonstrated extensive transcriptional heterogeneity during resistance development in EGFR-mutant NSCLC [9], while phosphoproteomic studies have revealed adaptive kinome remodeling associated with EGFR-TKI resistance [43]. Integrating exposome metrics with such multi-omic datasets may help identify exposure-linked signaling configurations and uncover novel therapeutic vulnerabilities.

### 10.3. Development of Exposome Biomarkers

A major barrier to studying exposure-associated resistance mechanisms is the difficulty of accurately quantifying environmental exposures across populations and over time, a challenge frequently highlighted in exposome research [15,16]. Objective exposure biomarkers may complement patient-reported histories and improve reproducibility across epidemiologic and translational studies [16,32]. Biomarkers reflecting tobacco and combustion exposure—including circulating cotinine levels, urinary metabolites of polycyclic aromatic hydrocarbons, and inflammatory cytokine signatures—have been proposed as measurable indicators of inhalational toxicant burden [35,73]. Oxidative stress markers and inflammation-associated transcriptional programs have been linked to chronic particulate exposure and pollutant-induced signaling activation in lung tissue [41,80]. Emerging evidence suggests that persistent environmental exposures may leave detectable epigenetic footprints, including DNA methylation patterns and chromatin remodeling signatures that reflect long-term exposure history [15,16]. The development of standardized exposome biomarker panels could facilitate integration of exposure assessment into prospective oncology studies and exposure-stratified clinical trials [32,33].

### 10.4. Longitudinal Resistance Modeling

Resistance to targeted therapy is increasingly recognized as a dynamic evolutionary process rather than a static endpoint, reflecting ongoing interactions between tumor cell populations and therapeutic selective pressures [2,14]. Longitudinal sampling strategies—including circulating tumor DNA analysis, serial biopsies, and repeated imaging—have therefore emerged as valuable approaches for monitoring clonal evolution and resistance development during treatment [12,13]. Recent genomic studies demonstrate that resistant tumor populations may arise through complex evolutionary trajectories involving multiple coexisting driver alterations within individual tumors [12]. In parallel, emerging research on drug-tolerant persister cells suggests that reversible transcriptional and epigenetic adaptations can precede the acquisition of stable resistance mutations [10,11]. Integrating longitudinal molecular monitoring with exposure-related data may therefore provide important insights into how environmental and occupational exposures influence resistance trajectories in oncogene-driven lung cancer.

### 10.5. Pharmacokinetic–Pharmacodynamic Studies

Environmental exposures may influence the efficacy of targeted therapy not only through signaling rewiring but also through pharmacokinetic mechanisms [105]. EGFR and ALK inhibitors are largely metabolized through cytochrome P450 pathways, particularly CYP3A enzymes, making systemic drug exposure potentially sensitive to environmental factors that modulate xenobiotic metabolism [2,89,105]. Inhalational toxicants such as cigarette smoke and combustion-derived pollutants can induce metabolic enzymes and inflammatory pathways that influence drug metabolism and transporter activity [16,41]. Consequently, exposure-related variability in CYP3A activity or efflux transporter expression (e.g., ABCB1) may alter effective intratumoral concentrations of tyrosine kinase inhibitors [47,93]. Future pharmacokinetic–pharmacodynamic studies integrating exposure biomarkers with drug concentration measurements and clinical outcomes may therefore clarify whether exposure-associated resistance arises primarily through signaling adaptation, altered drug disposition, or the interaction of both mechanisms.

### 10.6. Dedicated Studies in ALK-Rearranged NSCLC

Most available evidence linking environmental exposures to targeted therapy resistance derives from studies of EGFR-mutant lung cancer, whereas comparable data in ALK-rearranged NSCLC remain relatively limited [16,49]. Nevertheless, mechanistic studies indicate that adaptive signaling networks and receptor crosstalk contribute to the evolution of resistance in ALK-driven tumors [2,55]. Shared signaling pathways—including MAPK, PI3K/AKT, and MET-mediated bypass signaling—suggest that exposure-associated inflammatory and oxidative signaling may plausibly influence therapeutic variability in ALK-positive disease as well [35,47,49]. Future mechanistic and clinical studies examining exposure-related modulation of ALK-targeted therapy responses will therefore be essential to determine whether the exposome-driven resistance framework extends beyond EGFR-driven disease.

## 11. Conclusions

Resistance to EGFR- and ALK-targeted therapies has traditionally been interpreted primarily through a genomic lens. Converging experimental and epidemiologic evidence increasingly indicates that chronic respiratory exposures may modulate signaling plasticity and contribute to the evolution of adaptive resistance during targeted therapy.

Within this context, components of the respiratory exposome—including cigarette smoke, particulate air pollution, indoor combustion products, and occupational inhalational toxicants—activate inflammatory signaling, oxidative stress pathways, and adaptive kinase network responses relevant to lung tumor biology. These processes suggest that environmental exposures may represent an additional biological layer influencing treatment durability through context-dependent modulation of signaling networks.

The exposome-driven adaptive resistance framework proposed in this review should therefore be considered mechanistically plausible but not yet clinically established. Future prospective studies integrating structured exposure assessment with molecular profiling and longitudinal monitoring will be essential to determine the clinical relevance of exposure-informed precision oncology strategies.

## Figures and Tables

**Table 1:** Evidence linking respiratory exposures with signaling rewiring and targeted therapy outcomes in EGFR- and ALK-driven NSCLC (see abbreviations in footnote).

Exposure	Principal Biological Effects	Key Signaling Interfaces	Potential Clinical Implications for TKI Therapy	Representative Studies	Evidence Level
Active cigarette smoking	Oxidative stress; chronic inflammation; metabolic reprogramming	EGFR phosphorylation; Src/FAK activation; MET signaling bias; EMT induction	Shorter progression-free survival and reduced response to EGFR-TKIs in smokers	[26,27,28,35]	Moderate clinical and mechanistic evidence
Environmental tobacco smoke (ETS)	Low-level oxidative stress and inflammatory priming	EGFR activation; inflammatory cytokine signaling	Potential modulation of tumor signaling context and mutational landscape	[35,36]	Limited clinical evidence; mechanistic plausibility
Indoor combustion exposures (e.g., cooking fumes)	Particulate exposure; aldehydes and PAHs; epithelial injury	EGFR pathway modulation; oxidative and inflammatory signaling	Associated with lung cancer risk in never-smokers and distinct exposure patterns across populations	[5,36]	Predominantly epidemiologic evidence
Ambient air pollution (PM2.5, combustion particles)	ROS generation; macrophage recruitment; chronic inflammatory microenvironment	NF-κB, PI3K/AKT, STAT, and MAPK pathway activation	Potential promotion of tumor expansion and altered therapeutic vulnerability	[33,37]	Moderate mechanistic evidence
Occupational inhalational toxicants (silica, diesel exhaust, metal aerosols)	Persistent epithelial injury; inflammasome activation; cytokine signaling	NF-κB, MAPK, JAK/STAT signaling; adaptive kinome remodeling	Mechanistic plausibility for adaptive resistance via inflammatory and kinase signaling pathways	[38,39,40]	Preliminary mechanistic evidence
Composite respiratory exposome	Chronic inflammatory tone; oxidative stress; signaling network remodeling	Adaptive kinome activation; EMT induction; epigenetic plasticity	Potential contribution to heterogeneity in targeted therapy durability and resistance evolution	[6,9]	Hypothesis-generating

Abbreviations: EGFR, epidermal growth factor receptor; ALK, anaplastic lymphoma kinase; TKI, tyrosine kinase inhibitor; EMT, epithelial–mesenchymal transition; ROS, reactive oxygen species; NF-κB, nuclear factor kappa B; MAPK, mitogen-activated protein kinase; PI3K, phosphoinositide 3-kinase; AKT, protein kinase B; STAT, signal transducer and activator of transcription; PM2.5, particulate matter ≤ 2.5 µm; ETS, environmental tobacco smoke; PAHs, polycyclic aromatic hydrocarbons; FAK, focal adhesion kinase; Src, Src family kinases.

## Data Availability

All data from the first author and the corresponding author are available.

## Abbreviations

The following abbreviations are used in this manuscript:

ABCB1	ATP-Binding Cassette Subfamily B Member 1
ABCG2	ATP-Binding Cassette Subfamily G Member 2
AhR	Aryl Hydrocarbon Receptor
AKT	Protein Kinase B
ALK	Anaplastic Lymphoma Kinase
AMPK	AMP-Activated Protein Kinase
BBB	Blood–Brain Barrier
CNS	Central Nervous System
COX-2	Cyclooxygenase-2
CYP	Cytochrome P450
CYP3A4	Cytochrome P450 Family 3 Subfamily a Member 4
CYP3A5	Cytochrome P450 Family 3 Subfamily a Member 5
EGFR	Epidermal Growth Factor Receptor
EMT	Epithelial–Mesenchymal Transition
ERK	Extracellular Signal-Regulated Kinase
ETS	Environmental Tobacco Smoke
FAK	Focal Adhesion Kinase
FGFR	Fibroblast Growth Factor Receptor
IL	Interleukin
IL-1β	Interleukin-1 Beta
JAK	Janus Kinase
KRAS	Kirsten Rat Sarcoma Viral Oncogene Homolog
LKB1	Liver Kinase B1
MAPK	Mitogen-Activated Protein Kinase
MET	Mesenchymal–Epithelial Transition Receptor
NF-κB	Nuclear Factor Kappa B
NLRP3	NOD-Like Receptor Family Pyrin Domain Containing 3
NSCLC	Non-Small Cell Lung Cancer
PAHs	Polycyclic Aromatic Hydrocarbons
PI3K	Phosphoinositide 3-Kinase
PM2.5	Particulate Matter with Aerodynamic Diameter ≤2.5 µm
PXR	Pregnane X Receptor
ROS	Reactive Oxygen Species
RTK	Receptor Tyrosine Kinase
Src	Src Family Kinases
STAT	Signal Transducer and Activator of Transcription
TKI	Tyrosine Kinase Inhibitor
VEGF	Vascular Endothelial Growth Factor
WNT	Wingless/Integrated Signaling Pathway
